# Supplementary figures and images for: A novel IMU-based clinical assessment protocol for Axial Spondyloarthritis: a protocol validation study
Source: PeerJ. 2021 Jan 26;9:e10623. doi: 10.7717/peerj.10623 (PMC7845531; doi:10.7717/peerj.10623)

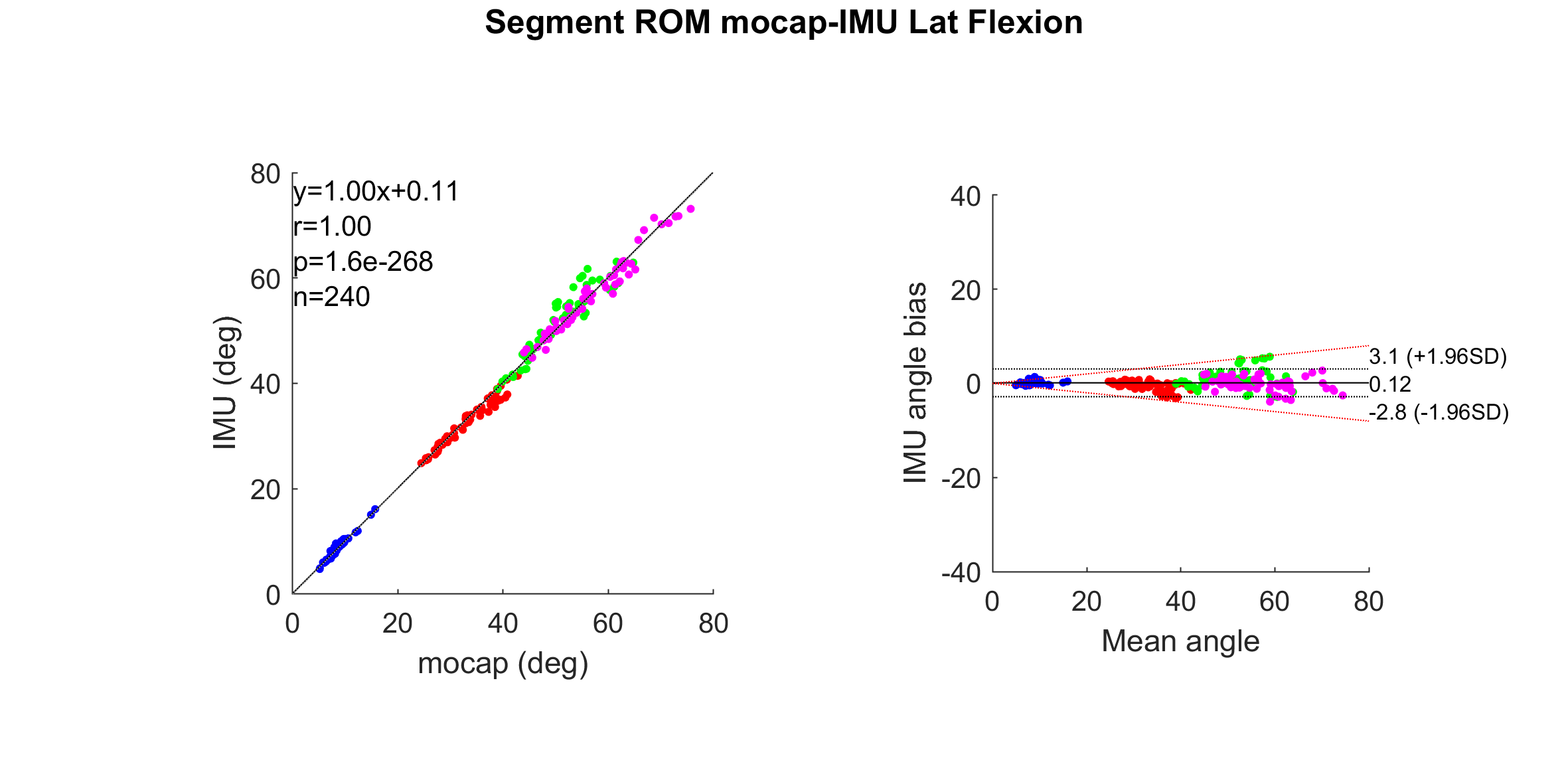

Supplement: Supplemental Information 1 [file peerj-09-10623-s001.zip › Funcs validation paper Luca Franco/QUAL_AVA_analysis/Relative ROM mocap-IMU_Lat_Flexion.png]

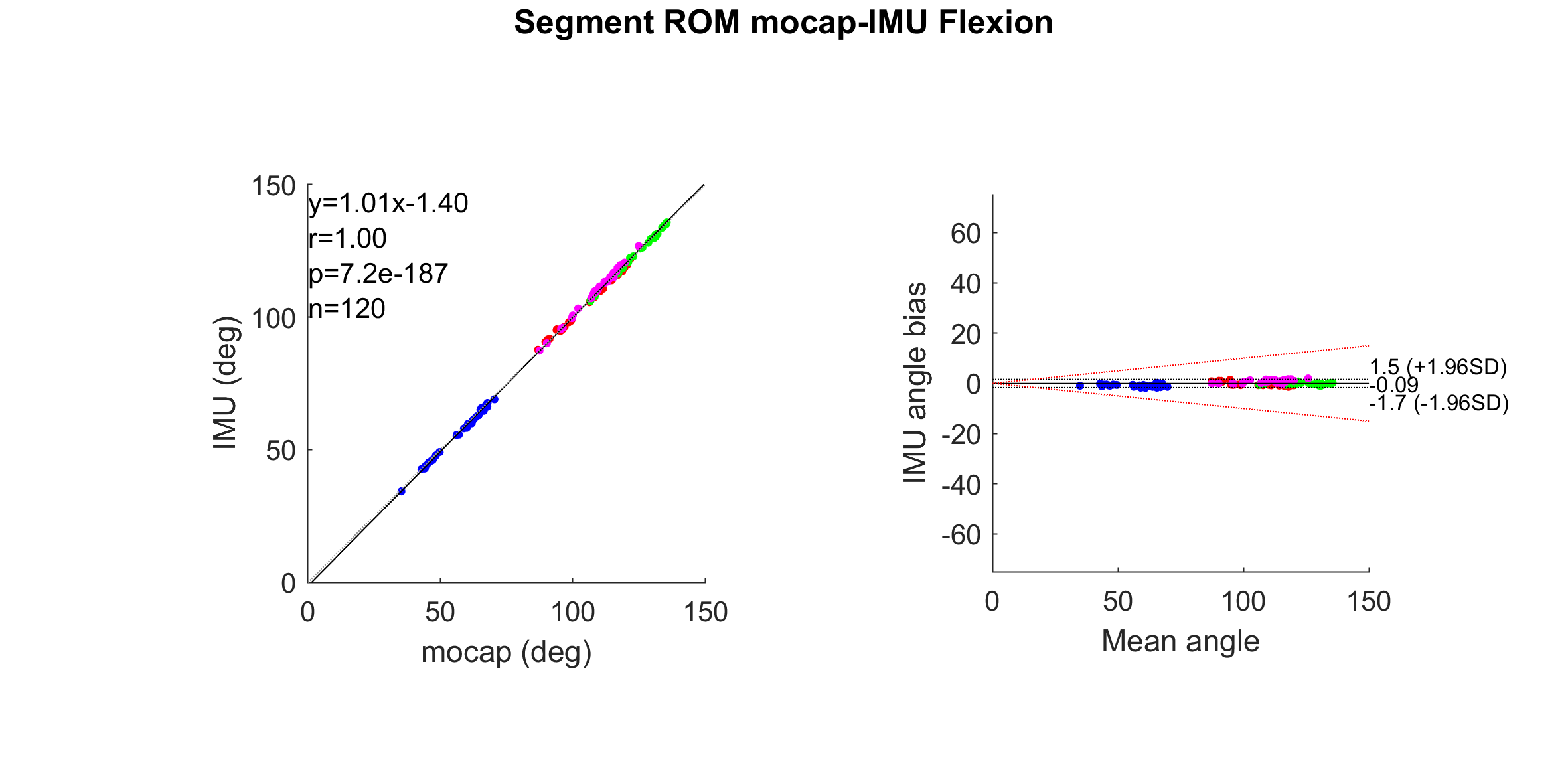

Supplement: Supplemental Information 1 [file peerj-09-10623-s001.zip › Funcs validation paper Luca Franco/QUAL_AVA_analysis/Relative ROM mocap-IMU_Flexion.png]

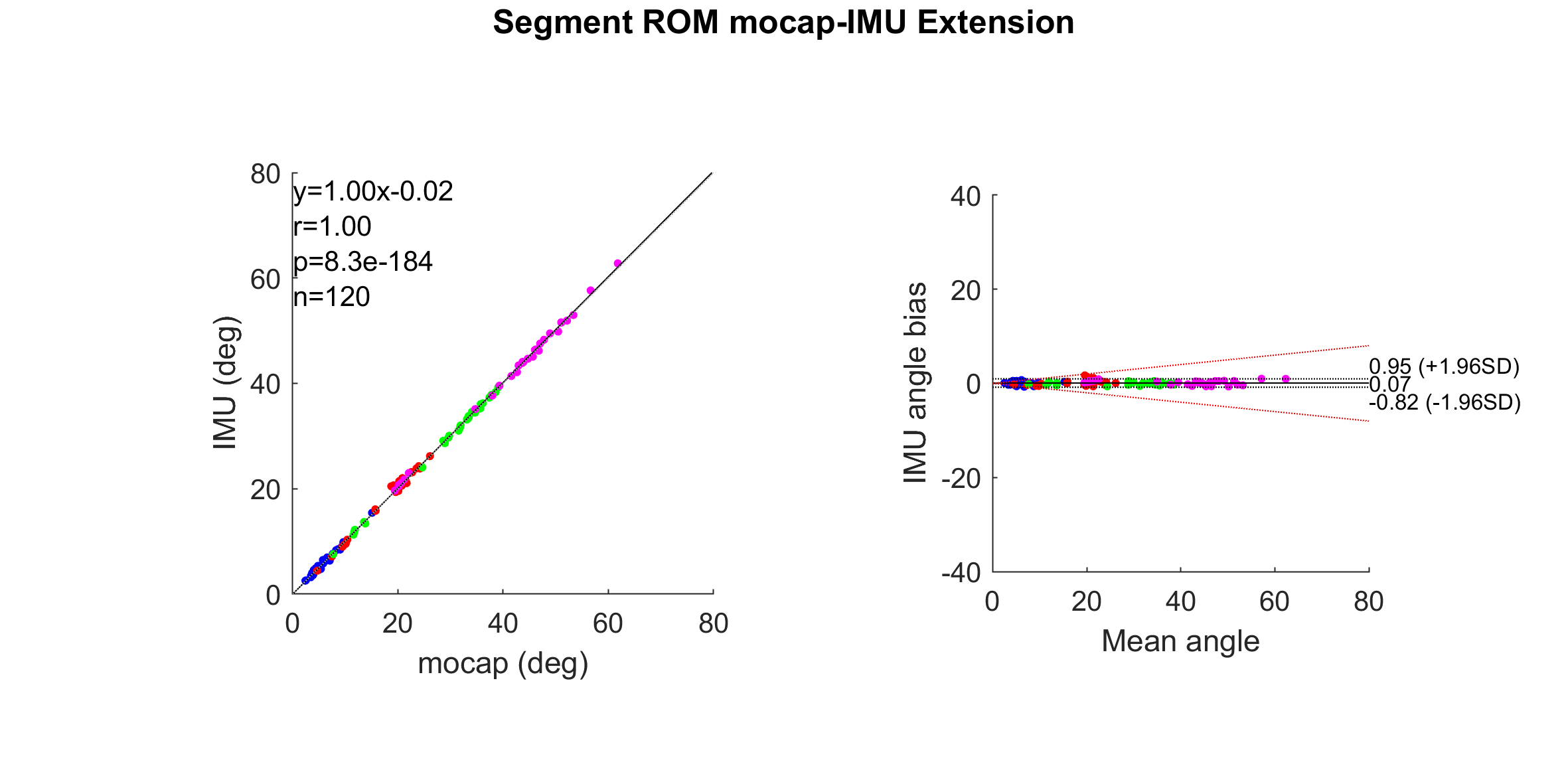

Supplement: Supplemental Information 1 [file peerj-09-10623-s001.zip › Funcs validation paper Luca Franco/QUAL_AVA_analysis/Relative ROM mocap-IMU_Extension.png]

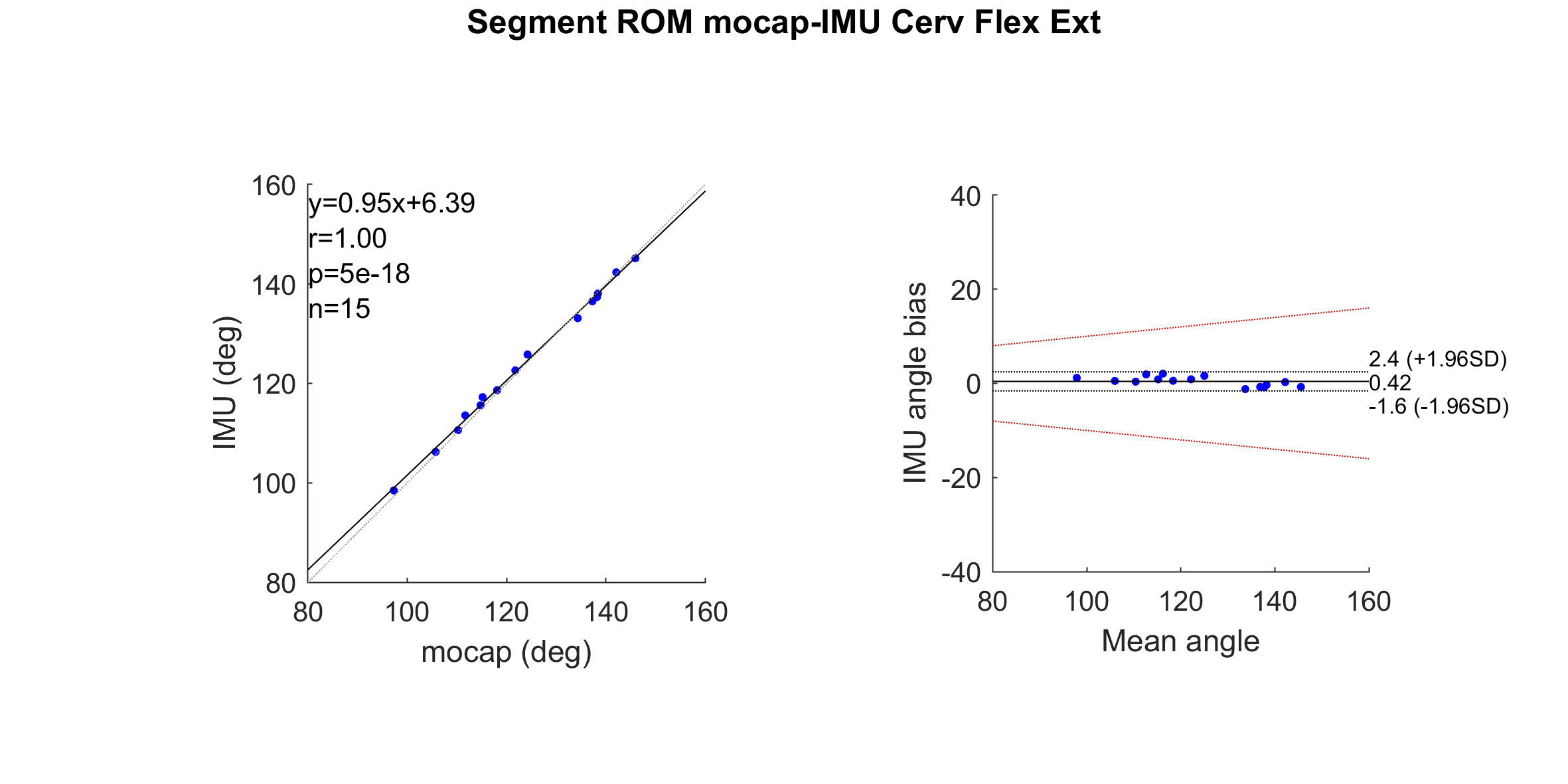

Supplement: Supplemental Information 1 [file peerj-09-10623-s001.zip › Funcs validation paper Luca Franco/QUAL_AVA_analysis/Relative ROM mocap-IMU_Cerv_FE.png]

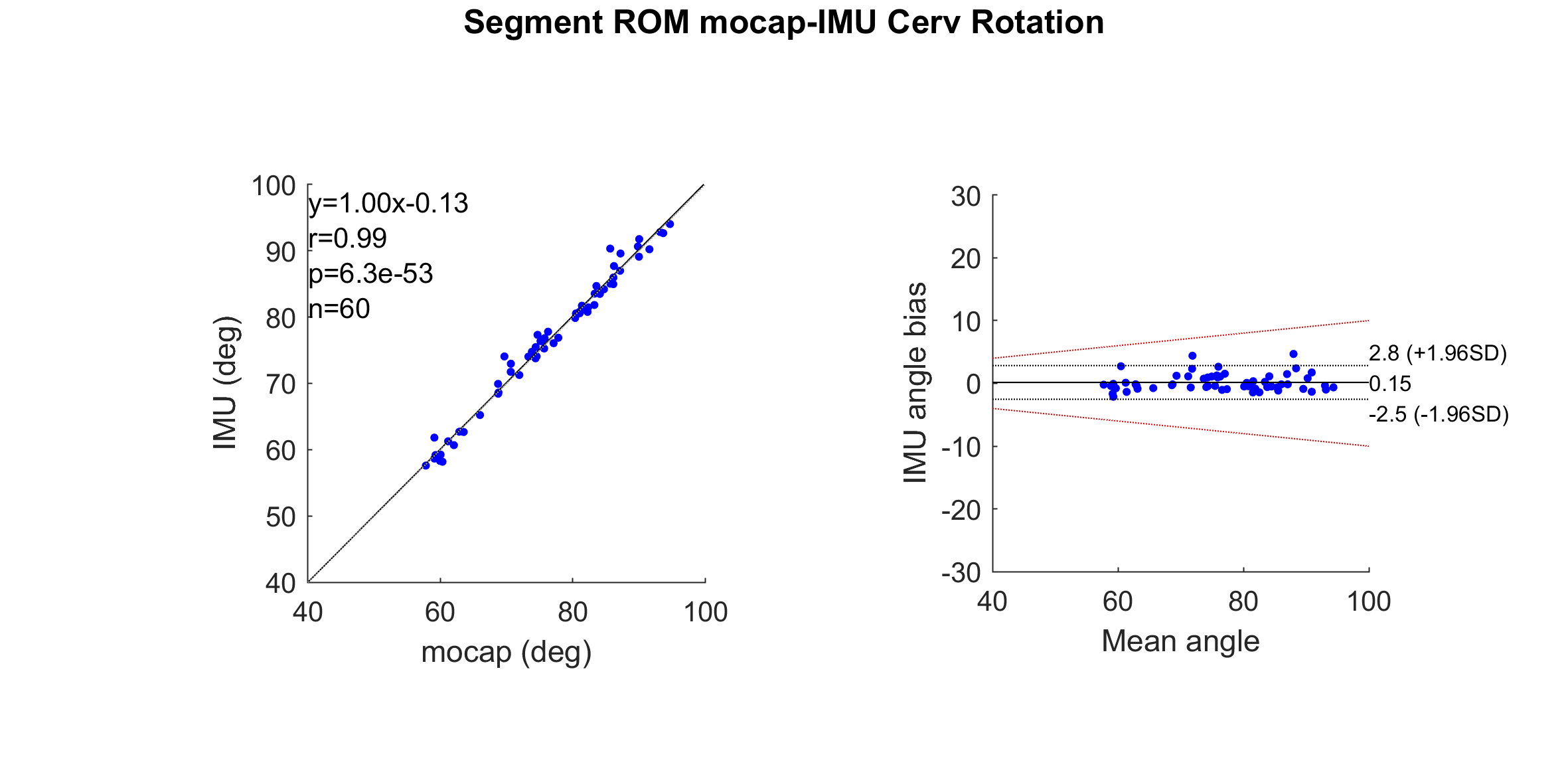

Supplement: Supplemental Information 1 [file peerj-09-10623-s001.zip › Funcs validation paper Luca Franco/QUAL_AVA_analysis/Relative ROM mocap-IMU_Cerv_Rotation.png]

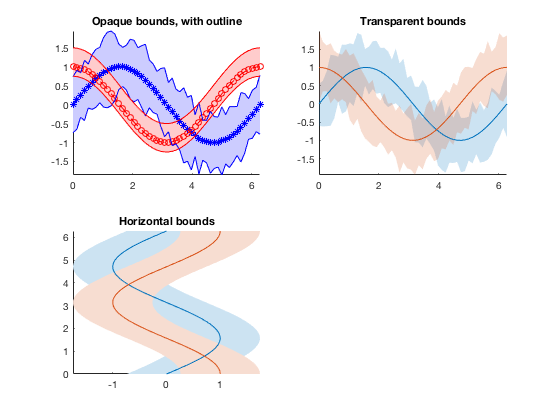

Supplement: Supplemental Information 1 [file peerj-09-10623-s001.zip › Funcs validation paper Luca Franco/kakearney-boundedline-pkg-50f7e4b/readmeExtras/README_03.png]

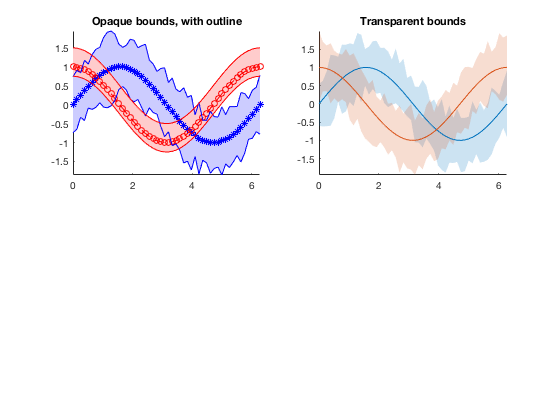

Supplement: Supplemental Information 1 [file peerj-09-10623-s001.zip › Funcs validation paper Luca Franco/kakearney-boundedline-pkg-50f7e4b/readmeExtras/README_02.png]

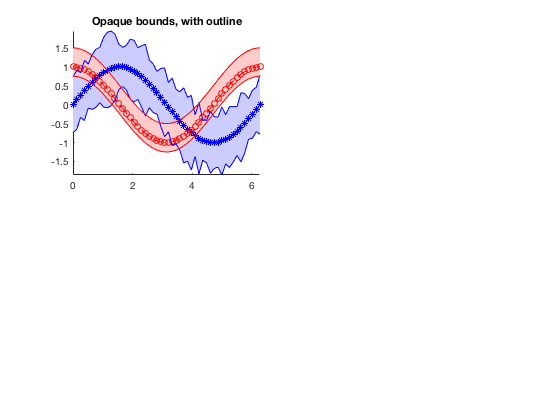

Supplement: Supplemental Information 1 [file peerj-09-10623-s001.zip › Funcs validation paper Luca Franco/kakearney-boundedline-pkg-50f7e4b/readmeExtras/README_01.png]

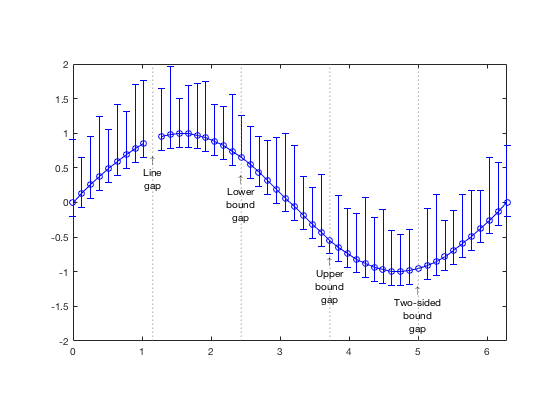

Supplement: Supplemental Information 1 [file peerj-09-10623-s001.zip › Funcs validation paper Luca Franco/kakearney-boundedline-pkg-50f7e4b/readmeExtras/README_05.png]

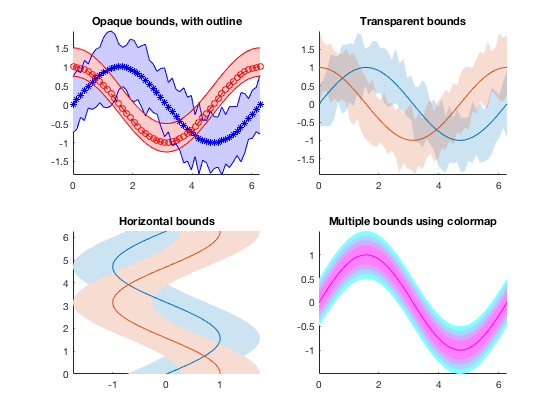

Supplement: Supplemental Information 1 [file peerj-09-10623-s001.zip › Funcs validation paper Luca Franco/kakearney-boundedline-pkg-50f7e4b/readmeExtras/README_04.png]

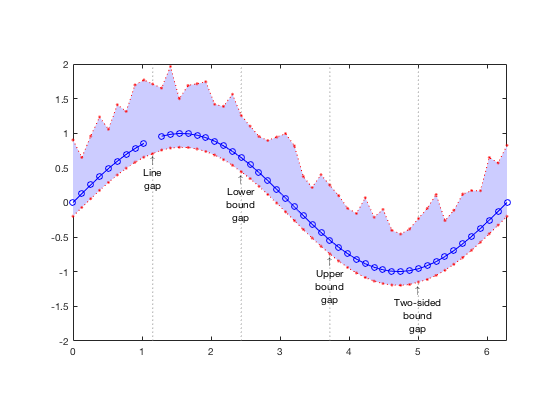

Supplement: Supplemental Information 1 [file peerj-09-10623-s001.zip › Funcs validation paper Luca Franco/kakearney-boundedline-pkg-50f7e4b/readmeExtras/README_06.png]

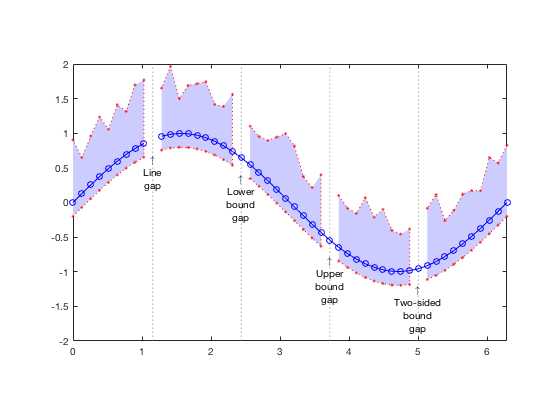

Supplement: Supplemental Information 1 [file peerj-09-10623-s001.zip › Funcs validation paper Luca Franco/kakearney-boundedline-pkg-50f7e4b/readmeExtras/README_07.png]

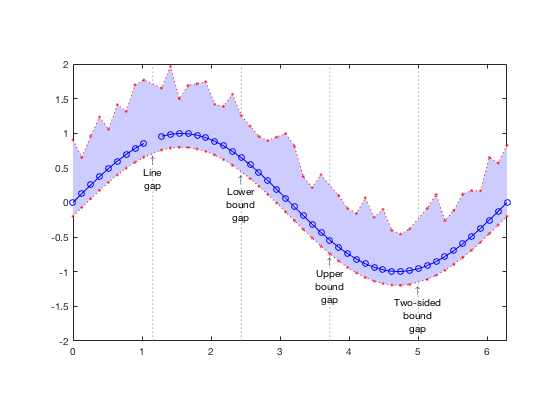

Supplement: Supplemental Information 1 [file peerj-09-10623-s001.zip › Funcs validation paper Luca Franco/kakearney-boundedline-pkg-50f7e4b/readmeExtras/README_08.png]

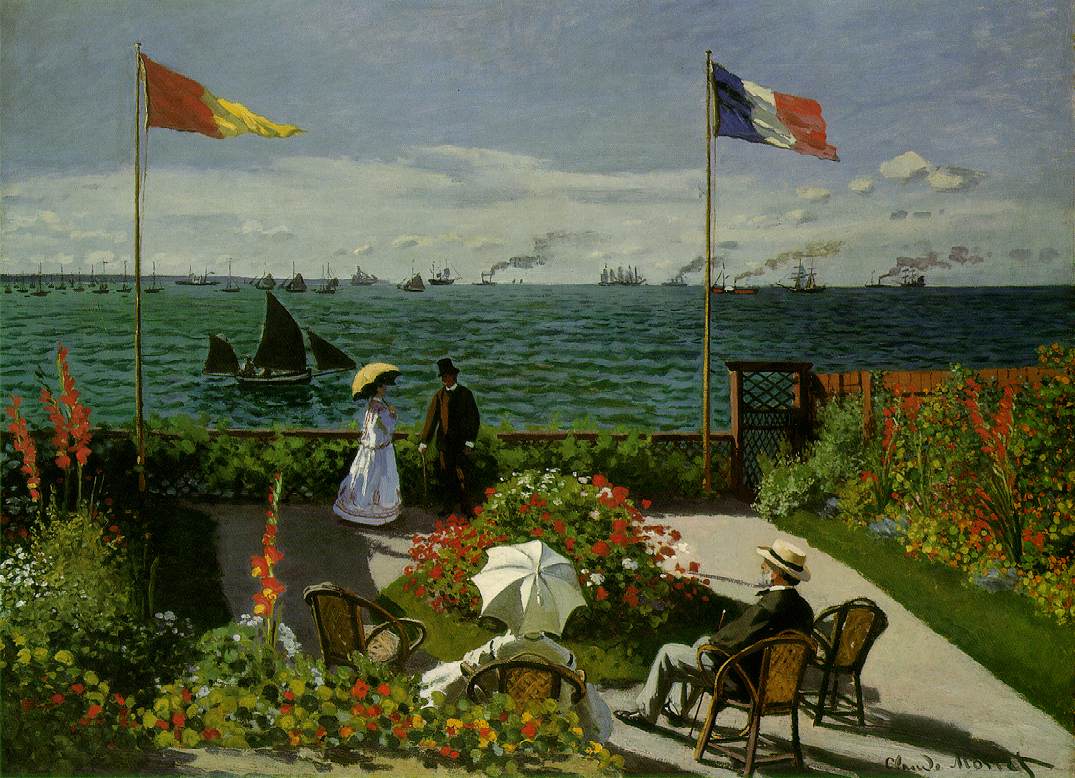

Supplement: Supplemental Information 1 [file peerj-09-10623-s001.zip › Funcs validation paper Luca Franco/kakearney-boundedline-pkg-50f7e4b/Inpaint_nans/monet_adresse.jpg]

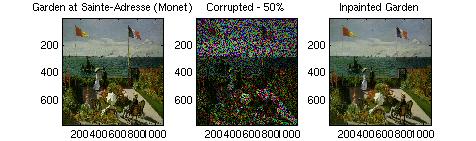

Supplement: Supplemental Information 1 [file peerj-09-10623-s001.zip › Funcs validation paper Luca Franco/kakearney-boundedline-pkg-50f7e4b/Inpaint_nans/garden50.jpg]

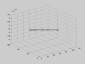

Supplement: Supplemental Information 1 [file peerj-09-10623-s001.zip › Funcs validation paper Luca Franco/kakearney-boundedline-pkg-50f7e4b/Inpaint_nans/demo/html/inpaint_nans_demo.png]

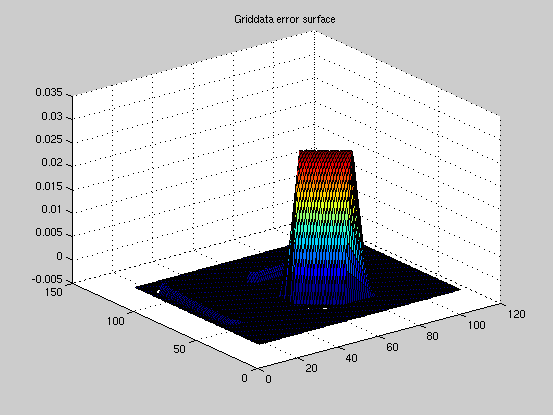

Supplement: Supplemental Information 1 [file peerj-09-10623-s001.zip › Funcs validation paper Luca Franco/kakearney-boundedline-pkg-50f7e4b/Inpaint_nans/demo/html/inpaint_nans_demo_05.png]

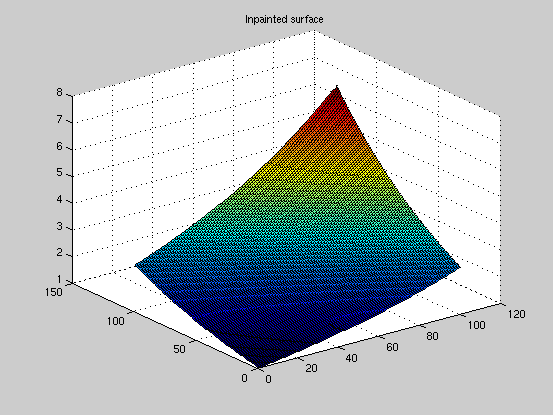

Supplement: Supplemental Information 1 [file peerj-09-10623-s001.zip › Funcs validation paper Luca Franco/kakearney-boundedline-pkg-50f7e4b/Inpaint_nans/demo/html/inpaint_nans_demo_04.png]

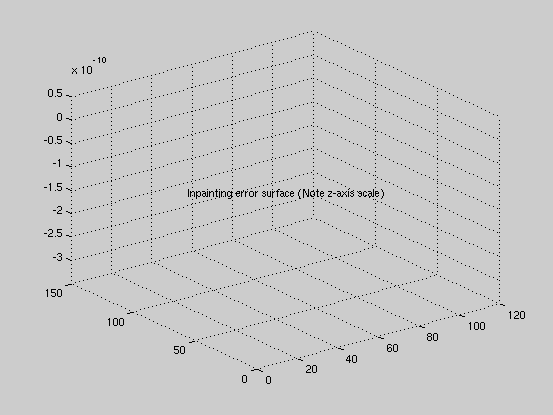

Supplement: Supplemental Information 1 [file peerj-09-10623-s001.zip › Funcs validation paper Luca Franco/kakearney-boundedline-pkg-50f7e4b/Inpaint_nans/demo/html/inpaint_nans_demo_06.png]

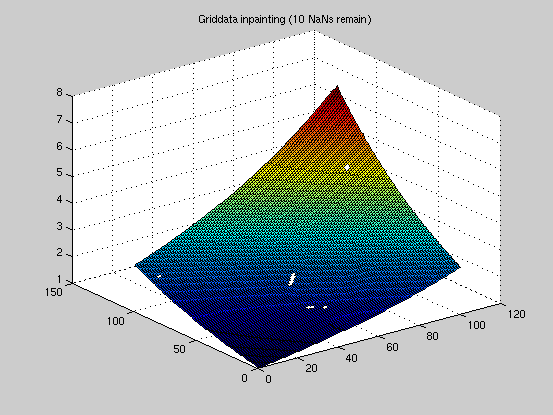

Supplement: Supplemental Information 1 [file peerj-09-10623-s001.zip › Funcs validation paper Luca Franco/kakearney-boundedline-pkg-50f7e4b/Inpaint_nans/demo/html/inpaint_nans_demo_03.png]

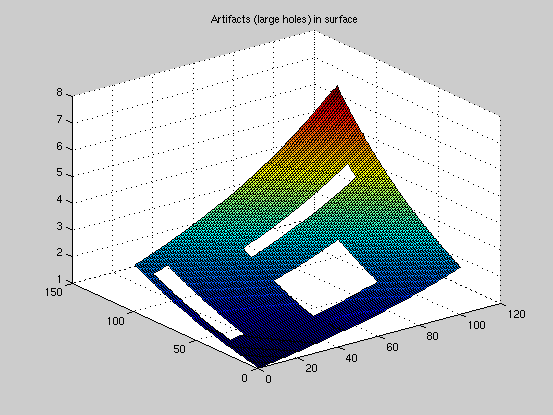

Supplement: Supplemental Information 1 [file peerj-09-10623-s001.zip › Funcs validation paper Luca Franco/kakearney-boundedline-pkg-50f7e4b/Inpaint_nans/demo/html/inpaint_nans_demo_02.png]

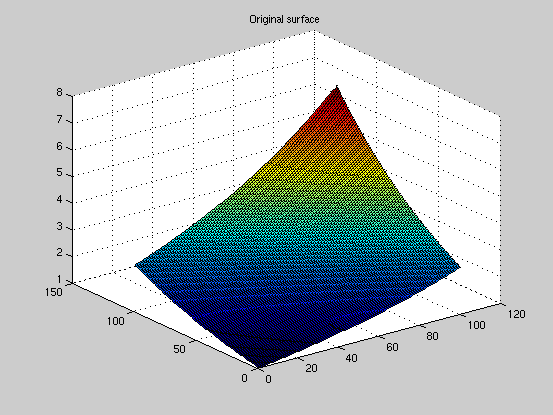

Supplement: Supplemental Information 1 [file peerj-09-10623-s001.zip › Funcs validation paper Luca Franco/kakearney-boundedline-pkg-50f7e4b/Inpaint_nans/demo/html/inpaint_nans_demo_01.png]
